# AACFlow: an end-to-end model based on attention augmented convolutional neural network and flow-attention mechanism for identification of anticancer peptides

**DOI:** 10.1093/bioinformatics/btae142

**Published:** 2024-03-07

**Authors:** Shengli Zhang, Ya Zhao, Yunyun Liang

**Affiliations:** School of Mathematics and Statistics, Xidian University, Xi'an 710071, China; School of Mathematics and Statistics, Xidian University, Xi'an 710071, China; School of Science, Xi’an Polytechnic University, Xi'an 710048, China

## Abstract

**Motivation:**

Anticancer peptides (ACPs) have natural cationic properties and can act on the anionic cell membrane of cancer cells to kill cancer cells. Therefore, ACPs have become a potential anticancer drug with good research value and prospect.

**Results:**

In this article, we propose AACFlow, an end-to-end model for identification of ACPs based on deep learning. End-to-end models have more room to automatically adjust according to the data, making the overall fit better and reducing error propagation. The combination of attention augmented convolutional neural network (AAConv) and multi-layer convolutional neural network (CNN) forms a deep representation learning module, which is used to obtain global and local information on the sequence. Based on the concept of flow network, multi-head flow-attention mechanism is introduced to mine the deep features of the sequence to improve the efficiency of the model. On the independent test dataset, the ACC, Sn, Sp, and AUC values of AACFlow are 83.9%, 83.0%, 84.8%, and 0.892, respectively, which are 4.9%, 1.5%, 8.0%, and 0.016 higher than those of the baseline model. The MCC value is 67.85%. In addition, we visualize the features extracted by each module to enhance the interpretability of the model. Various experiments show that our model is more competitive in predicting ACPs.

## 1. Introduction

ACPs are a special kind of antimicrobial peptides (AMPs) that can kill cancer cells. In the treatment of cancer, compared with traditional radiotherapy, chemotherapy, targeted therapy, and other programs, ACPs only kill diseased cells, do not damage normal cells, and have no side effects on the human body (Liao *et al.* 2018). This is a safer and more effective treatment for cancer patients and can reduce the risk of subsequent chemotherapy (Xu *et al.* 2018). At present, researchers have found and confirmed the anticancer function of some peptides. Cationic antimicrobial peptides (CAPs) are positively charged and amphiphilic and can destroy cancer cells by membrane dissolution (Chung *et al.* 2019, Hermant *et al.* 2021). Host defense peptides (HDPs) found in plants and amphibians can recognize cancer cells in lung cancer, melanoma, and breast cancer, and overcome their drug resistance (Silva *et al.* 2018). The polypeptide D-K6L9 also has anticancer activity due to electrostatic interaction with phosphatidylserine exposed on the surface of cancer cells (Huang *et al.* 2011). While all of these ACPs offer potential alternatives for cancer treatment, only a few have made it to clinical trials, so the discovery of more effective ACPs remains urgent.

ACPs usually consist of 5–50 amino acids. At present, many sequencing technologies have used traditional feature representation methods to determine whether a peptide has anticancer activity. Schaduangrat *et al.* (2019) used classical feature representation methods (amino acid composition (AAC), dip-eptide composition (DPC), and physicochemical property (PCP)) to characterize the sequence, and used support vector machines (SVM) and random forests (RF) as classifiers to identify the sequence. Boopathi *et al.* (2019) also used seven manual features (AAC, DPC, amino acid index (AAIF), quasi-sequence-order (QSO), composition-transition-distribution (CTD), conjoint triad (CTF), binary profile (NC5)) and SVM to construct the model, mACPpred. Grisoni *et al.* (2019) applied artificial neural networks to identify ACPs. Rao *et al.* (2020) designed a multi-view feature extraction model called ACPred-Fuse, which employed a total of 29 different sequence-based feature encoding methods and used RF to further select features. You *et al.*’s (2022) GRCI-Net model used binary structure and K-mer sparse matrix to extract the features of peptide sequences, and used principal component analysis to fuse the two features. The output was then fed into a classifier composed of bidirectional long short-term memory network (Bi-LSTM) and CNN. Wan *et al.* (2021) selected AAC, N5C5, k-space, and position-specific scoring matrix (PSSM) as feature representation methods and used SVM and sequential minimal optimization (SMO) for prediction. Zhou *et al.* (2022) developed ACP_MS using monMonoKGap and K-mer to represent peptide sequences and AdaBoost to extract the optimal set of features. Yuan *et al.* (2023) proposed ACP-OPE, which uses the position information of peptide sequences and several traditional hand-crafted feature methods (AAC, DPC, the composition of k-spaced amino acid group pairs (CKSAAGP), K-mer) to represent peptide sequences. The ensemble model of Bi-LSTM, CNN, and light gradient boosting machine algorithm (Light-GBM) is used as the classifier.

However, these traditional manual feature extraction methods are designed based on all kinds of peptide sequences, which cannot fully express the sequence specificity of ACPs. Some of the above models use machine learning to select features, but in fact, machine learning algorithms have limited learning ability for long sequences, and it is difficult to extract deep features. In recent years, with the development of natural language processing technology, many researchers have applied deep representation learning to feature extraction of peptide sequences, such as Protein Feature Engineering Toolkit (ProFET) (Ofer and Linial 2015) and UDSMProt (Strodthoff *et al.* 2020). Lv *et al.* (2021) constructed IACP-DRLF, which employed two pre-trained sequence embedding models (soft symmetric alignment embedding and unified representation embedding) to convert peptide sequences into digital vectors, and used Light-GBM to determine the optimal feature space. However, because it used the feature representation model trained on other datasets and ignored the sequence specificity, the final prediction results were not ideal.

**Figure 1.**
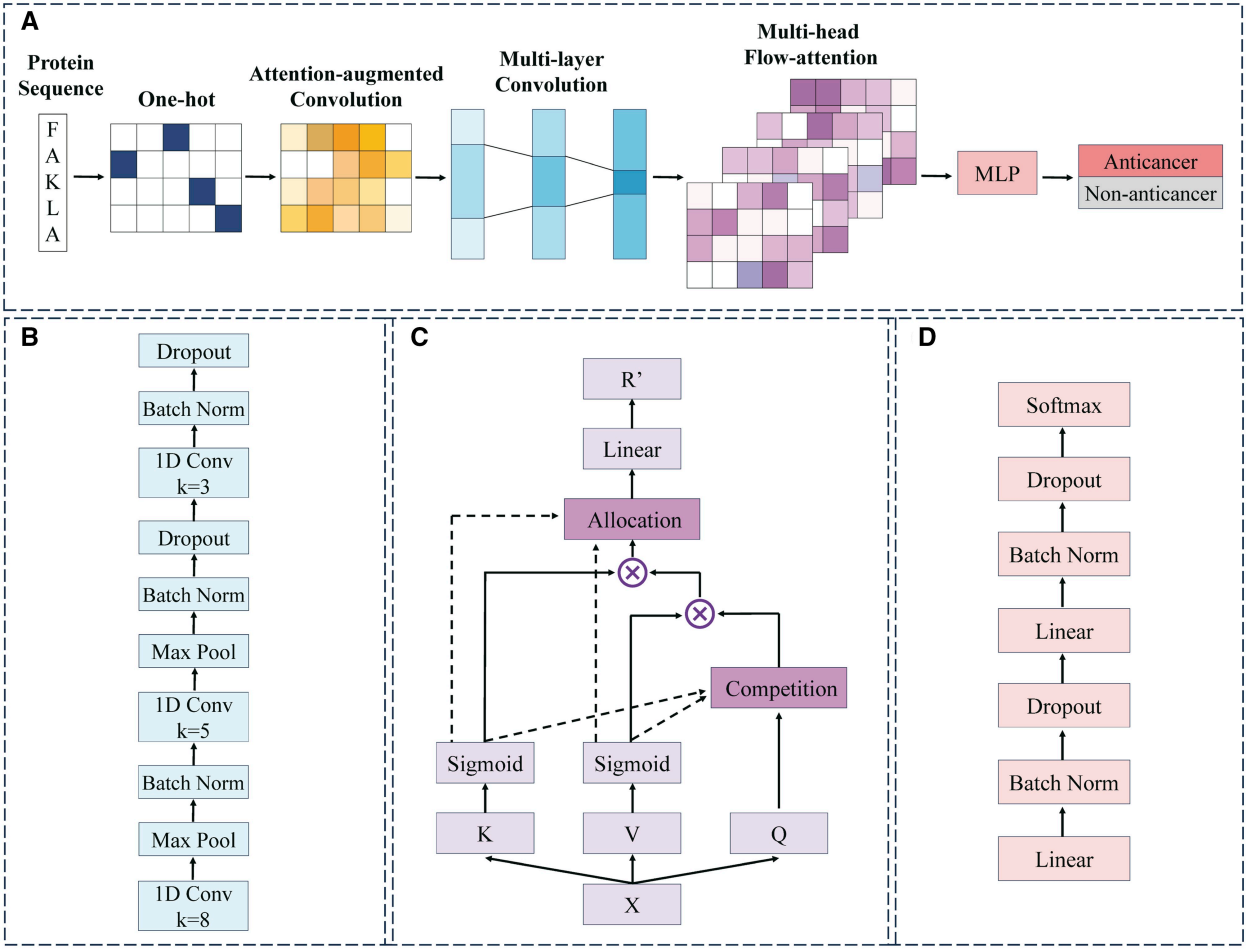
The architecture of AACFlow.

In this article, based on the analysis of the above studies, a novel end-to-end deep learning model, AACFlow, is proposed to identify ACPs. In this model, sequence-based deep representation learning method is used to extract sequence features. AAConv is used for the first time to obtain global and local features of peptide sequences, and multi-layer convolution neural networks are used to further fuse the two features. Compared with directly using pre-trained language models, our feature extraction method can learn specific features of ACPs and improve the prediction accuracy of the model. In addition, previous studies prefer to use Bi-LSTM to mine the deep features of ACPs, but the cyclic recursive algorithm of Bi-LSTM will occupy a lot of storage space and reduce the operation efficiency. Therefore, this article introduces a special multi-head flow-attention mechanism instead of Bi-LSTM to extract high-level features. Flow-attention mechanism can capture the dependencies between distal features in the sequence, and is also suitable for parallel training to improve the training efficiency of the model. Finally, we use multi-layer perceptron (MLP) to obtain the predicted probability of peptide sequences and achieve the final classification. The model structure is shown in Fig. 1.

## 2. Materials and methods

### 2.1. Dataset

To fairly compare our model with other models, we use the same dataset as in Yuan *et al.*’s (2023) study. The dataset can be directly downloaded from https://webs.iiitd.edu.in/raghava/anticp2/, all the data after experimental verification. The dataset contains 859 ACPs from the CancerPPD database (Tyagi *et al.* 2015). Meanwhile, to construct a balanced dataset, the same number of AMPs is randomly selected as non-ACPs. It is worth stating that the entire data do not contain peptides with both anticancer and antimicrobial properties. The dataset is randomly divided into training dataset and independent test dataset according to 8:2, and the number of positive and negative samples in each subset is 1:1, which is the same as in the baseline paper. All peptide sequences contain 4–50 residues. The details of the datasets are shown in Table 1.

**Table 1.** Details of the datasets.

Datasets	Positive	Negative	Total
Training	688	688	1376
Independent	171	171	342

### 2.2. Overview of AACFlow

**Figure 2.**
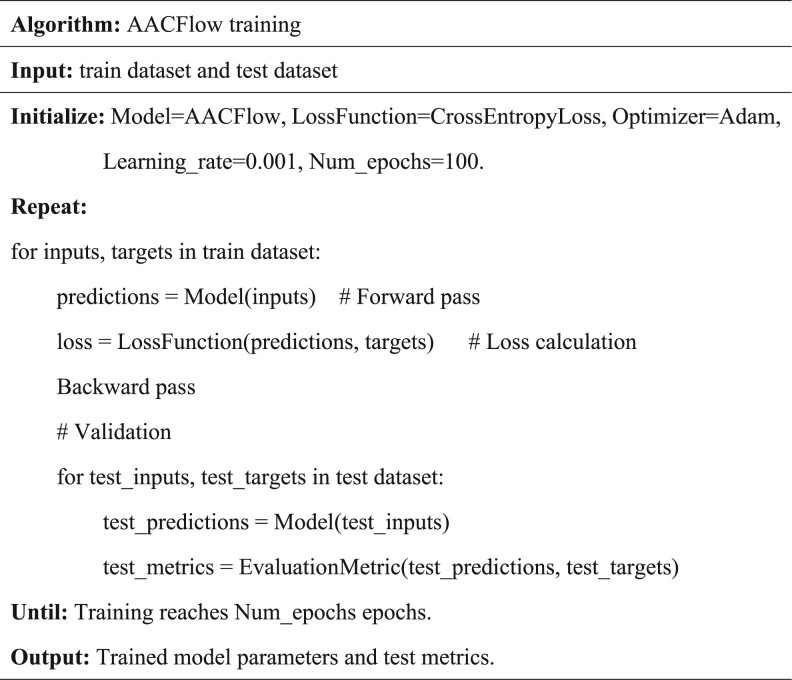
The pseudocode for model training.

The input of the end-to-end model AACFlow is the original peptide sequence and the outputs are the probabilities that the peptide sequence is an anticancer peptide and a non-anticancer peptide, respectively. The model integrates the shallow feature extraction module, the deep feature extraction module, and the classification module in turn, which can avoid the error accumulation between different modules in the training process. The specific process of AACFlow is shown in Fig. 1a. The shallow feature extraction module is a deep representation learning module composed of one-hot encoding, AAConv layer, and multi-layer convolution neural networks. One-hot encoding can completely represent the original peptide sequence, AAConv layer can extract the global and local features of the sequence, and multi-layer convolution module can use convolution kernels of different sizes to achieve feature fusion. Fig. 1b shows the detailed structure of multi-layer convolution module. Multi-head flow-attention mechanism is a deep feature extraction module, which can discover the interactions between distant features with less computation. Its principle is shown in Fig. 1c. The classification module consists of fully connected layers, batch normalization layers, and dropout layers, as shown in Fig. 1d. The training process of the model and the parameters of the main layers are shown in Figure 2 and Table 2.

**Table 2.** The parameters of AACFlow.

Layers	Parameters
AAConv	Out channels = 100, kernel size = 10, num heads = 1
CNN-1	Out channels = 32, kernel size = 8
CNN-2	Out channels = 48, kernel size = 5
Dropout-1	Dropout ratio = 0.2
CNN-3	Out channels = 96, kernel size = 3
Dropout-2	Dropout ratio = 0.2
Flow-attention	Hid dim = 44, num heads = 4, dropout ratio = 0.05
Linear-1	Out features = 256
Dropout-3	Dropout ratio = 0.2
Linear-2	Out features = 48
Dropout-4	Dropout ratio = 0.2
Linear-3	Out features = 2, activation = “softmax”

### 2.3. Deep representation learning module

Deep representation learning module can automatically learn global and local features of the sequence (Wang *et al.* 2021, Lv *et al.* 2021, Wang *et al.* 2022). Traditional manual feature extraction methods are constructed according to AAC, evolutionary information, sequence information, physical and chemical properties, and so on (Shi *et al.* 2022, Li *et al.* 2022, Zhang and Jing 2023). Although the characteristics of peptide sequences can be obtained from many aspects, the original sequence itself cannot be retained, and some subtle deep information existing in the sequence may be ignored. We adopt one-hot encoding (Novic and Randic 2008) to transform the original sequence into a numeric vector without artificially adding any other information. One-hot encoding uses 21-bit status registers to represent 21 amino acids (20 common amino acids and “O” (the letter that complements the sequence)), that is, 21-dimensional binary vectors are used to represent each amino acid, which can maintain the original appearance of the sequence to the greatest extent (Czerniecka *et al.* 2016). For example, A is represented as [1, 0, 0, 0, 0, 0, 0, 0, 0, 0, 0, 0, 0, 0, 0, 0, 0, 0, 0, 0, 0], and D is represented as [0, 1, 0, 0, 0, 0, 0, 0, 0, 0, 0, 0, 0, 0, 0, 0, 0, 0, 0, 0, 0]. Then, the original vector is fed into deep neural network to automatically learn the shallow and abstract features of the sequence.

There is no doubt that CNN has powerful feature extraction ability, but it is only suitable for extracting local features of the sequence, while the global features of the sequence are also crucial for identifying ACPs. Therefore, we use AAConv layer to extract sequence features, which can overcome this defect of CNN and use attention mechanism to capture the long-distance interaction information of the sequence (Zhang *et al.* 2022, Yu *et al.* 2023). The original feature *X* is fed into CNN layer and multi-head self-attention layer, respectively, and the formula is as follows:

(1) C(X)=Conv(X),

(2) $$O_{h}=\mathrm{Softmax}\left(\frac{\left(XW_{q}\right){\left(XW_{k}\right)}^{T}}{\sqrt{d_{k}^{h}}}\right)\left(XW_{v}\right),$$

(3) $$\mathrm{MHA}\left(X\right)=\mathrm{Concat}\left[O_{1},\ldots ,O_{Nh}\right]W^{O}.$$

*Conv*(.) stands for convolution operation. *O_h_* and *MHA*(*X*) are the outputs of single-head self-attention and multi-head self-attention, respectively. *Nh* is the number of attention heads. *W^O^*, *W_q_*, *W_k_*, and *W_v_* are learnable parameter matrices. $d_{k}^{h}$ is the depth of keys of the head *h* in MHA. Then, relu activation function is used to learn the nonlinear relationship between the two features, and the final output *AAConv*(*X*) is obtained. The formula is as follows:

(4) AAConv(X)=ReLU(Concat[C(X),MHA(X)]).

Multi-layer convolutional neural networks are used to further fuse the features obtained by AAConv layer. The features are scanned by windows with different sizes, which enhances the ability of CNN to capture different features (Ma *et al.* 2023, He *et al.* 2023). As shown in Fig. 1b, the size of convolution kernels gradually decreases with the deepening of CNN layers. A larger convolution kernel has a larger receptive field and can comprehensively consider more features. A smaller convolution kernel has a smaller receptive field, which can mine subtle changes between different features and prevent information loss. Meanwhile, we add batch normalization layers and dropout layers after CNN layers and maxpooling layers to improve the portability of the model.

### 2.4. Flow-attention mechanism

Self-attention mechanism can capture the dependencies between long-distance features and grasp the global information of the sequence (Liu *et al.* 2023). At the same time, it can also focus on the important regions of the sequence and mine the key features. The parallel training of multiple self-attention mechanisms can extract different types of features and enrich sequence information, but it will also increase the complexity of the model. To this end, we adopt flow-attention mechanism, which can effectively accelerate the training speed of the model while retaining the ability of attention mechanism to focus on the global and key information of the sequence (Yao *et al.* 2023, Xin *et al.* 2023). For a sequence of length *n*, the computational complexity of ordinary self-attention mechanism is *O*(*n*^2^), while the computational complexity of flow-attention mechanism is only *O*(*n*). Flow-attention mechanism introduces the competition and allocation mechanism according to the conservation law of flow network.

As shown in Fig. 1c, *Q*, *K*, and *V* vectors are first obtained by linear projection of the input *X*:

(5) Q=WqX,K=WkX,V=WvX,

where *W_q_*, *W_k_*, and *W_v_* are weight matrices. Then we map *Q* and *K* to the non-negative region:

(6) $$\varphi \left(Q\right)=\mathrm{Sigmoid}\left(Q\right)=\frac{1}{1+e^{-Q}},$$

(7) $$\varphi \left(K\right)=\mathrm{Sigmoid}\left(K\right)=\frac{1}{1+e^{-K}}.$$

According to the conservation principle, the input information flow equals the output information flow. The competition or allocation mechanism is introduced when information flow is limited. The input information flow at the sink is *I*, which can be expressed as follows:

(8) $$I=\varphi \left(Q\right)\sum_{j=1}^{m}\varphi {\left(K_{j}\right)}^{T}.$$

When the input information flow remains constant, the competition mechanism is introduced at the source, and the amount of information that the attention mechanism interacts with the external network is set to 1 to form the inflow conservation. In this case, the output information of the source is *O′*:

(9) $$O^{\prime }=\varphi \left(K\right)\sum_{i=1}^{n}\frac{\varphi {\left(Q_{i}\right)}^{T}}{I_{i}},$$

which is the amount of information provided by the source. The formula for the competition mechanism is as follows:

(10) $$V^{\prime }=\mathrm{Softmax}(O^{\prime })⊗V,$$

where ⊗ refers to elementwise multiplication. The output information flow of the source is *O*, which can be expressed as follows:

(11) $$O=\varphi \left(K\right)\sum_{i=1}^{n}\varphi {\left(Q_{i}\right)}^{T},$$

When it remains constant, the allocation mechanism is introduced at the sink, and the amount of information that the attention mechanism interacts with the external network is set to 1, which is called the outflow conservation of the source. Then, the inflow information at the sink is *I′*:

(12) $$I^{\prime }=\varphi \left(Q\right)\sum_{j=1}^{m}\frac{\varphi {\left(K_{j}\right)}^{T}}{O_{j}},$$

which represents the amount of information received at the sink. The formula for the allocation mechanism is as follows:

(13) $$R=\mathrm{Sigmoid}(I^{\prime })⊗[\frac{\varphi \left(Q\right)}{I}(\varphi {\left(K\right)}^{T}V^{\prime })],$$

where *R* is the attention score. Finally, the output feature *R'* is obtained by linear transformation. In this study, we obtain 94 × 64 dimensional deep features.

### 2.5. MLP module

The deep features *R*' extracted above are input to MLP module for the final identification. As shown in Fig. 1d, there are three linear layers, in which the number of neurons is gradually reduced. The last linear layer uses softmax function to obtain the predicted probabilities. In addition, batch normalization layers and dropout layers are used to improve the robustness of the model.

### 2.6. Model evaluation

In this study, the model is trained by five-fold cross validation on the training dataset, and then the performance of this model is tested on the independent test dataset. We use the following four evaluation metrics that are the same as those in the benchmark paper (Yuan *et al.* 2023): accuracy (ACC), sensitivity (Sn), specificity (Sp), Matthew’s correlation coefficient (MCC), and area under ROC curve (AUC) (Manavalan *et al.* 2019, Wang *et al.* 2020, Ma *et al.* 2022). ACC is a fundamental evaluation metric in classification problems, which indicates the proportion of samples that the model predicts correctly. Sn, also known as true positive rate, is used to measure the ability of a model to correctly predict positive samples. Sp is also known as true negative rate, which is used to measure model's ability to predict negative samples. MCC describes the correlation between the actual result and the predicted result. ROC curve and AUC evaluate the quality of binary classification model as a whole. ROC curve is plotted using two metrics: true positive rate and false positive rate, and AUC is the area between ROC curve and its horizontal axis. A higher AUC value indicates a better model. The formulas are as follows:

(14) ACC=Sp++Sn+Sp++Sn++Sp−+Sn−,Sn=Sp+Sp++Sn−,Sp=Sn+Sp−+Sn+,MCC=Sp+×Sn+−Sp−×Sn−(Sp++Sn−)(Sp++Sp−)(Sn++Sp−)(Sn++Sn−),

where $S_{p}^{+}$, $S_{n}^{+}$, $S_{p}^{-}$, and $S_{n}^{-}$ represent correctly predicted positive samples, negative samples, incorrectly predicted positive samples, and negative samples, respectively.

## 3. Results and discussion

### 3.1. Analysis of sequences and features

**Figure 3.**
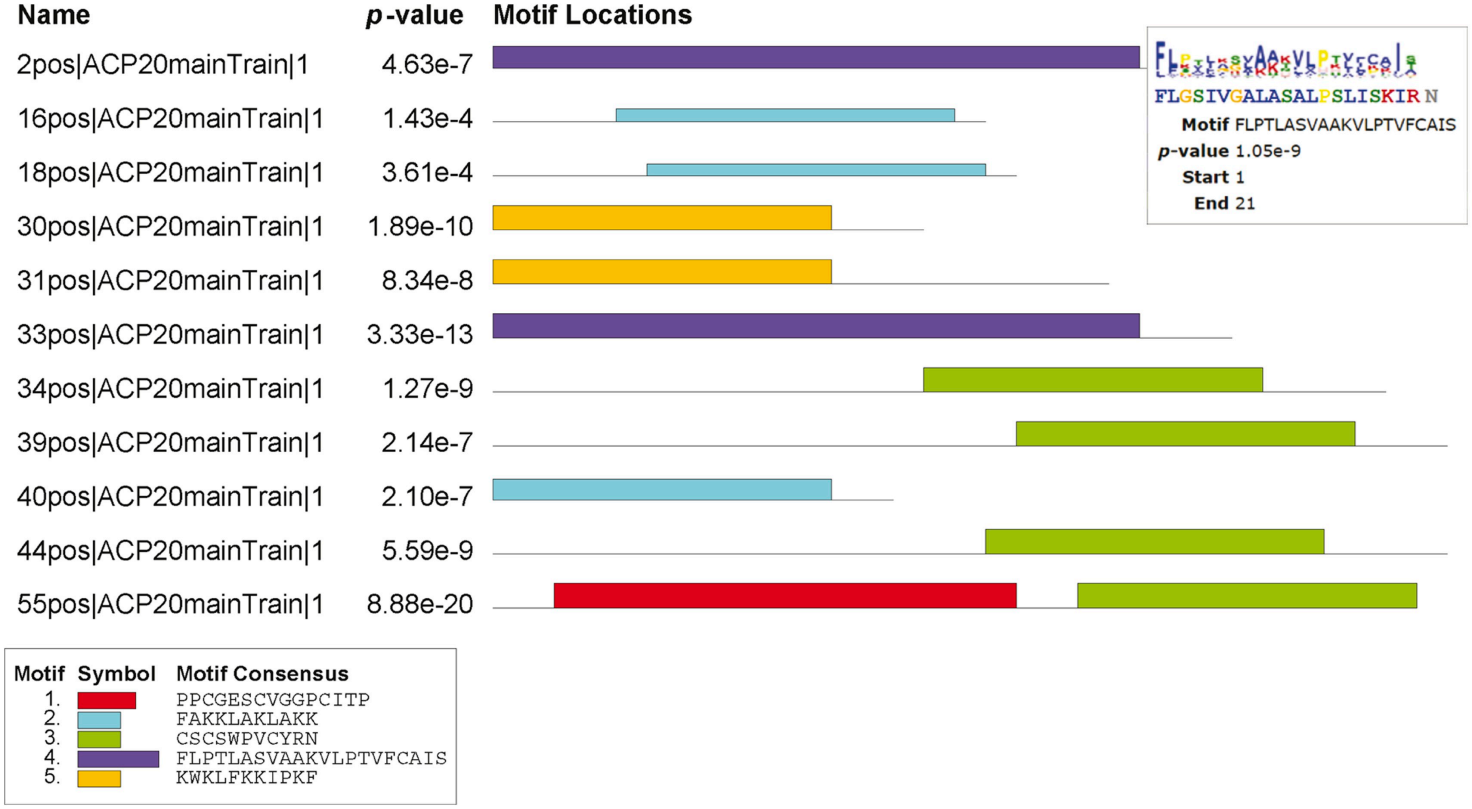
Detailed distribution information of motifs in partial ACPs.

Before building the identification model of ACPs, we analyze the sequence characteristics of ACPs in the training dataset. Motifs are short sequences with a certain structure or function that can be used to identify and classify specific genes or proteins, so finding motifs in the sequence that has anticancer functions is crucial for identifying ACPs. MEME 5.5.0 (MEME—Submission form (meme-suite.org)) is a website for finding motifs that are widespread in biological sequences (Bailey and Elkan 1994). In the training dataset, a total of 190 sequences have obvious motifs except the excessively short sequences, of which 100 sequences have motifs in the N-terminal, 2 sequences have motifs in the C-terminal, 54 sequences have motifs in the middle, 2 sequences have motifs throughout the whole sequence, 3 sequences have motifs in the N-terminal and middle, and 29 sequences have motifs in the middle and C-terminal. In addition, more than 85% of the sequences have motifs whose length is more than half of the entire sequence length. Figure 3 shows the distribution of motifs in some representative ACPs in the training dataset. The second column is the combined match *p*-value, which is defined as the probability that a random sequence would have position *p*-values such that the product is smaller or equal to the value calculated for the sequence under test. The rightmost column shows the location of motif sites. The black horizontal lines represent the entire sequence, and the colored rectangular blocks represent the different motifs and their positions in the sequence. The details of the motif for the first sequence are shown in the upper left box. A smaller position *p*-value indicates a more important motif. The sequence is 22-bp, and the motif starts at position 1 and ends at position 21. It can be seen that the motifs of ACPs are all over the N-terminal, the middle, and the C-terminal of the whole sequence, and occupy a relatively large proportion.

Based on the above analysis, in order to find the key regions in the whole sequence and grasp global information, we introduce AAConv layer with the advantages of both CNN and attention mechanism, which can effectively avoid the separation of sequence information by local convolution operations. The results of ablation experiment on AAConv layer are shown in Table 3. When AAConv layer in the model is removed and local information of the sequence is obtained directly by multi-layer CNN module, the ACC, Sn, Sp, AUC, and MCC values on the training dataset are 89.82%, 88.89%, 90.67%, 0.9454, and 79.59%, respectively. On the independent test dataset, the ACC, Sn, Sp, AUC, and MCC values are reduced by 5.27%, 3.51%, 7.02%, 0.052, and 10.53%, respectively. It can be seen that adding AAConv layer to extract the global information of the sequence helps to improve the ability of the model to mine effective motifs.

**Table 3.** The results of ablation experiment on the AAConv layer.

Dataset	Model	ACC (%)	Sn (%)	Sp (%)	AUC	MCC (%)
Training	Non-AAConv	89.82	88.89	90.67	0.9454	79.59
	AACFlow	91.23	88.00	94.81	0.9519	82.72
Independent	Non-AAConv	78.65	79.53	77.78	0.8399	57.32
	AACFlow	83.92	83.04	84.80	0.8919	67.85

### 3.2. The effectiveness of flow-attention mechanism

**Figure 4.**
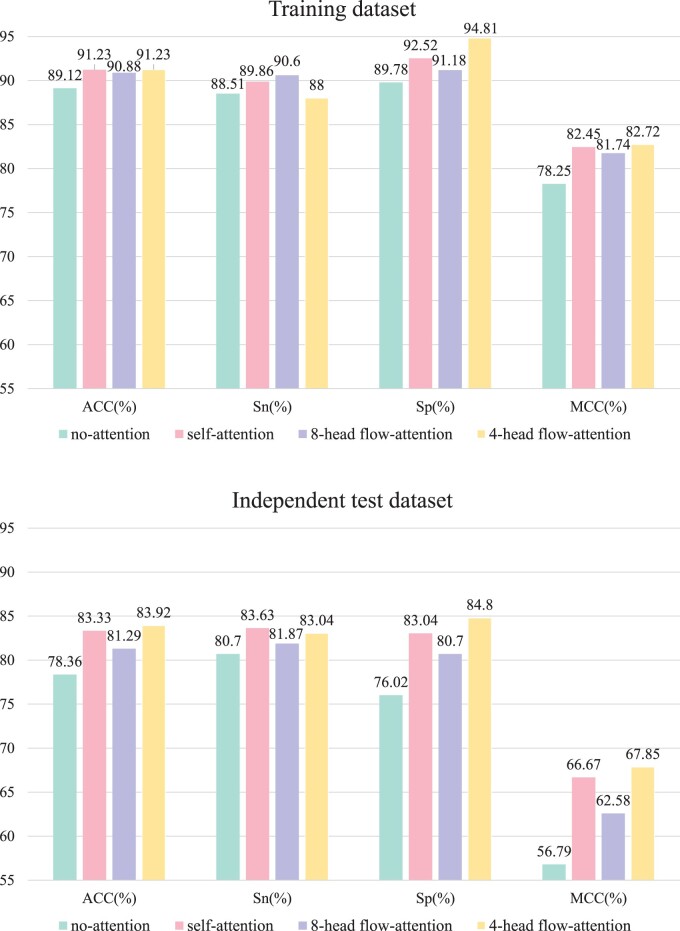
Analysis results of flow-attention mechanism.

**Figure 5.**
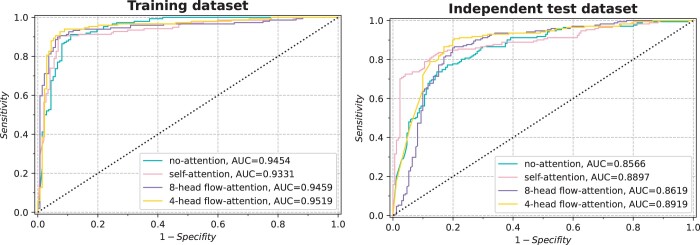
ROC curves of different attention mechanisms.

Self-attention mechanisms are favored by researchers because they can take into account long-distance interactions between features and thus improve prediction accuracy. However, the model with multi-head self-attention mechanism has quadratic complexity, and the performance of the model will be reduced when the sequence is longer. In this article, flow-attention mechanism is adopted to solve this problem effectively. To illustrate the advantages of flow-attention mechanism, we compare the two, and the results are shown in Figs 4 and 5. On the training dataset, the ACC values of self-attention mechanism and flow-attention mechanism are equal. For Sp, MCC, and AUC values, flow-attention mechanism is highest. The Sn value is only 1.86% lower than self-attention mechanism. The ACC, Sp, MCC, and AUC values of flow-attention mechanism are also the highest on the independent test dataset. In the process of model training, the minimum time for one iteration of flow-attention mechanism is 57.27 s, while self-attention mechanism needs 65.84 s. In addition, we also analyze the number of heads. It is usually set to 4 or 8. When the number of heads is set to 8, the ACC, Sn, Sp, MCC, and AUC values on the training dataset are 90.88%, 90.60%, 91.18%, 81.74%, and 0.9459, respectively, and the ACC, Sn, Sp, MCC, and AUC values on the independent test dataset are 81.29%, 81.87%, 80.70%, 62.58%, and 0.8619, respectively. The overall performance of the model is lower than AACFlow. Therefore, we finally adopt 4-head flow-attention mechanism.

### 3.3. Comparison with existing methods

Our model adopts deep representation learning method to comprehensively extract low-level features of the sequence, and uses multi-head flow-attention mechanism to mine high-level features. This makes up for the shortcomings of traditional manual feature extraction methods in previous studies that cannot fully represent the hidden information of the sequence, and it is also friendly when computer memory resources are limited. For a large number of deformation sequences, AACFlow also has good identification ability. To prove that our model is effective, it is compared with other existing models on independent test datasets, and the results are shown in Table 4. The ACC and Sp values of AACFlow are 83.9% and 84.8%, respectively, which are 4.9% and 8% higher than the existing best model ACP-OPE (Yuan *et al.* 2023). The Sn value is 83%, second only to AntiCP (Tyagi *et al.* 2013) and ACPred (Schaduangrat *et al.* 2019). However, the other evaluation metrics of these two models are very low, indicating that their overall performance is far inferior to AACFlow. Under the premise that the similarity between the independent test dataset and the training dataset is extremely small, AACFlow still achieves ideal results, which indicates that the model is superior and portable in predicting ACPs.

**Table 4.** Comparison with existing methods on the independent test dataset.

Model	ACC (%)	Sn (%)	Sp (%)
AACFlow	83.9	83.0	84.8
ACP-OPE	79.0	81.5	76.8
iACP-DRLF	77.5	80.7	74.3
AntiCP_2.0	75.4	77.5	73.4
AntiCP	50.6	100	12.0
ACPred	53.5	85.6	21.4
ACPred-FL	44.8	67.1	22.5
ACPpred-Fuse	68.9	69.2	68.6
PEPred-Suite	53.5	33.1	73.8
iACP	55.1	77.9	33.2

### 3.4. Visualization of different features

In order to visually demonstrate the ability of each module in AACFlow to extract different sequence features, we employ t-distributed stochastic neighbor embedding technology (t-SNE) (Fang *et al.* 2023) to reduce features from high dimensions to 2 dimensions and visualize them. T-SNE is a nonlinear dimensionality reduction method, which maps the probability distribution between high-dimensional objects to low-dimensional space by affine transformation. The results are shown in Fig. 6. Figures A, B, 100, 500, and E represent one-hot coding features, shallow features extracted by AAConv layer, features fused by multi-layer CNN module, deep features extracted by flow-attention mechanism, and prediction probabilities obtained by MLP module, respectively. On the training dataset, the positive and negative samples encoded by one-hot coding are mixed. In the AAConv layer, the features of most positive samples can be perfectly captured and two clusters are vaguely formed. After multi-layer CNN module, there is an obvious boundary between positive and negative samples. Then, the powerful flow-attention mechanism extracts key features that are easy to distinguish, and gathers positive and negative samples on the left and right sides, respectively. MLP module further screens samples, reducing the number of positive samples that are misclassified. Similarly, on the independent test dataset, our model can also mine discriminative features and separate them. This shows that every part of the model is indispensable, which can extract beneficial features and improve the identification ability of the model.

**Figure 6.**
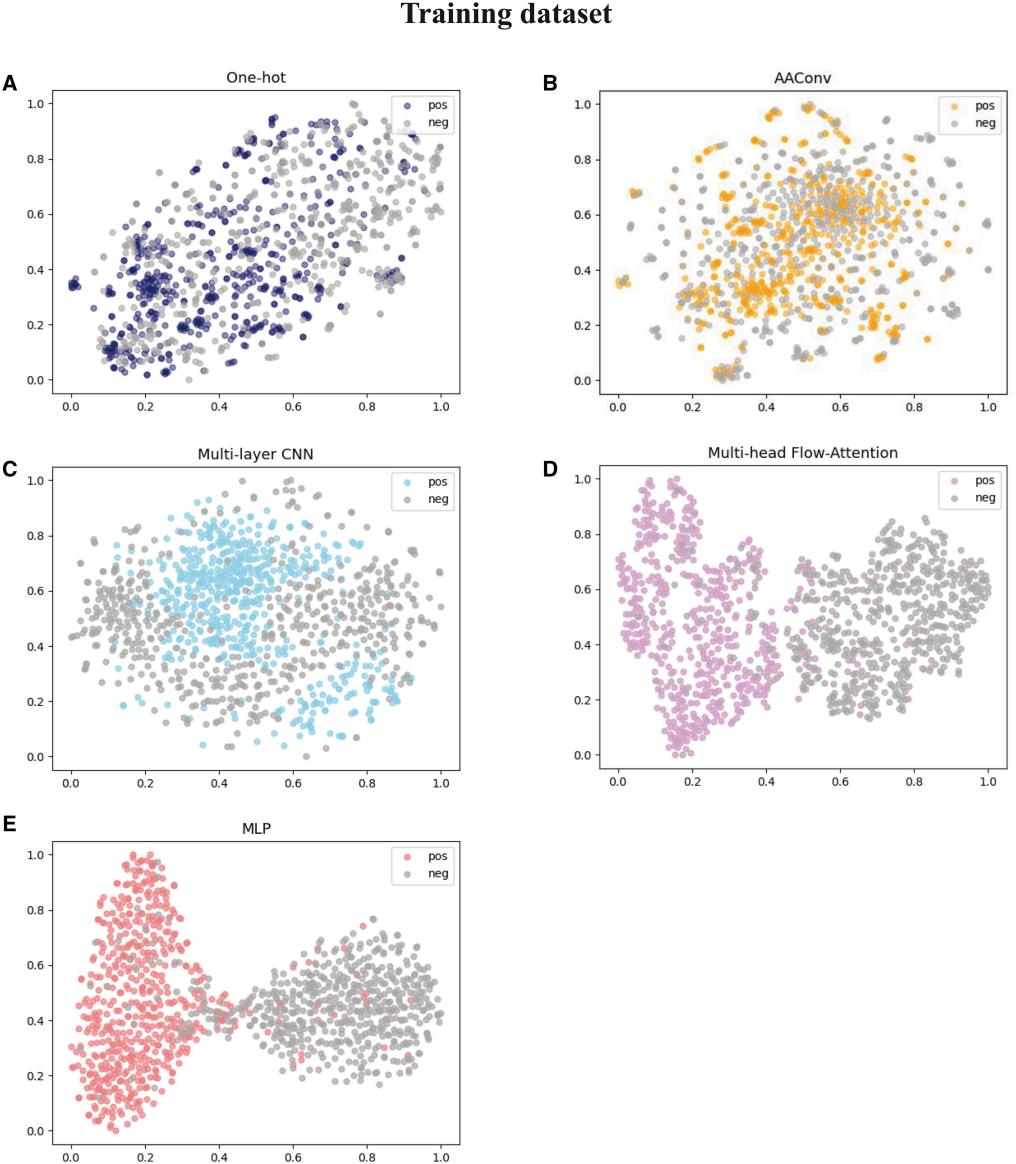
Visualization of the features of the different modules.

**Figure 6.**
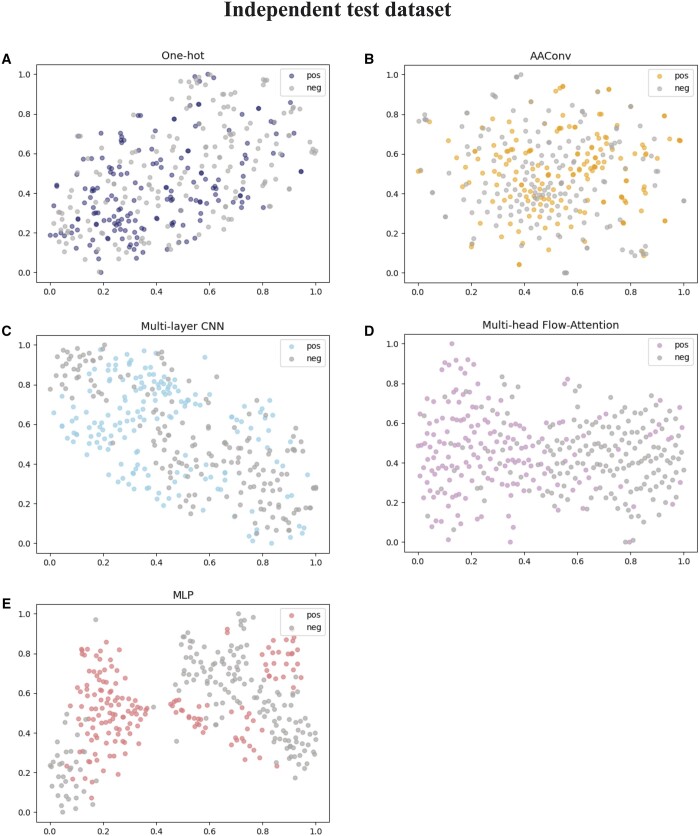
Continued

## 4. Program installation and use guide

The benchmark datasets and source codes used in this study are available on GitHub at https://github.com/z11code/AACFlow. The folder “data” contain the training dataset and the independent test dataset. If you want to use our model, first you need to install the following programs: python = 3.8, torch = 1.8.0, pandas = 1.1.3, and numpy = 1.18.0. In addition, it is also desirable to convert the dataset files to csv format. Then you can run model.py on the training dataset in the ‘code’ folder and save the model. model.py contains the parts of AACFlow. predict.py is used to test the performance of the model, and its inputs are the independent test dataset and Model.pt. Model.pt is the best model we have ever trained, and you can even use it to make predictions on your own datasets.

## 5 Conclusions

ACPs are natural biological inhibitors for the treatment of cancer, so compared with other treatment much safer. However, ACPs that can be used in biological experiments are limited, and in the face of a large number of protein deformation sequences, it is urgent to develop an identification model of ACPs with high accuracy. Based on the end-to-end training method, we build a model, AACFlow. Using AAConv layer to learn shallow features of ACPs can avoid the loss of global information compared with using only CNN layers in previous studies. Then, flow-attention mechanism is used to capture the further interaction information between sequence features. Flow-attention mechanism has the characteristics of low computational complexity and small storage space. A series of experiments show that the overall prediction performance of the proposed model is improved. Moreover, feature visualization results affirm the contribution of each part in the model to the whole. In conclusion, AACFlow can help biologists and medical professionals conduct in-depth research on ACPs and solve cancer problems as early as possible. However, the accuracy of the model is still lower than 90%, so in the future work, we will continue to improve the model to enhance the identification performance.

## Data Availability

The codes and datasets are accessible at https://github.com/z11code/AACFlow.
